# Prevalence, awareness, treatment and control of hypertension and their determinants: results from a national survey in Kenya

**DOI:** 10.1186/s12889-018-6052-y

**Published:** 2018-11-07

**Authors:** Shukri F. Mohamed, Martin K. Mutua, Richard Wamai, Frederick Wekesah, Tilahun Haregu, Pamela Juma, Loise Nyanjau, Catherine Kyobutungi, Elijah Ogola

**Affiliations:** 1 Health and Systems for Health Unit, African Population and Health Research Center (APHRC), Nairobi, Kenya; 2 Division of Health Sciences, Warwick Medical School, University of Warwick, Coventry, UK; 3 Department of Cultures, Societies and Global Studies, North Eastern University, Massachusetts, USA; 4 Julius Global Health, Julius Center for Health Sciences and Primary Care, University Medical Center, Utrecht University, Utrecht, Netherlands; 5 Division of Non Communicable Diseases, Ministry of Health, Nairobi, Kenya; 6 Clinical Medicine, University of Nairobi, Nairobi, Kenya

**Keywords:** Hypertension, Awareness, Treatment, Control, Non-communicable diseases, Sub-Saharan Africa, Africa, Kenya

## Abstract

**Background:**

Hypertension is the most important risk factor for cardiovascular diseases and the leading cause of death worldwide. Despite growing evidence that the prevalence of hypertension is rising in sub-Saharan Africa, national data on hypertension that can guide programming are missing for many countries. In this study, we estimated the prevalence of hypertension, awareness, treatment, and control. We further examined the factors associated with hypertension and awareness.

**Method:**

We used data from the 2015 Kenya STEPs survey, a national cross-sectional household survey targeting randomly selected people aged 18–69 years. Demographic and behavioral characteristics as well as physical measurements were collected using the World Health Organization’s STEPs Survey methodology. Descriptive statistics were used to estimate the prevalence, awareness, treatment and control of hypertension. Multiple logistic regression models were used to identify the determinants of hypertension and awareness.

**Results:**

The study surveyed 4485 participants. The overall age-standardized prevalence for hypertension was 24.5% (95% confidence interval (CI) 22.6% to 26.6%). Among individuals with hypertension, only 15.6% (95% CI 12.4% to 18.9%) were aware of their elevated blood pressure. Among those aware only 26.9%; (95% CI 17.1% to 36.4%) were on treatment and 51.7%; (95% CI 33.5% to 69.9%) among those on treatment had achieved blood pressure control. Factors associated with hypertension were older age (*p* < 0.001), higher body mass index (BMI) (*p* < 0.001) and harmful use of alcohol (*p* < 0.001). Similarly, factors associated with awareness were older age (*p* = 0.013) and being male (*p* < 0.001).

**Conclusion:**

This study provides the first nationally-representative estimates for hypertension in Kenya. Prevalence among adults is high, with unacceptably low levels of awareness, treatment and control. The results also reveal that men are less aware of their hypertension status hence special attention should focus on this group.

## Background

As the primary contributor to cardiovascular disease (CVD), hypertension (or high blood pressure) is the leading cause of morbidity and mortality worldwide [1, 2]. Globally, hypertension, which is defined as systolic blood pressure (SBP) ≥ 140 mmHg or diastolic blood pressure (DBP) ≥ 90 mmHg, or taking antihypertensive medication [3], is estimated to be responsible for 9.4 million deaths, about 17% of the total deaths in 2012 and 7% of total disability adjusted life years (DALYs) [4]. Worldwide, the overall prevalence of hypertension in adults aged 18 years and above was 24% for men and 20% in women in 2015, rising from 594 million to 1.13 billion in 1975 and 2015 respectively [5].

While in Africa hypertension was considered non-existent early in the twentieth century [6], sub-Saharan Africa (SSA) has the highest prevalence in the world today reaching approximately 30% as reported in a recent systematic review and meta-analysis [7]. As high as 54.1% mean prevalence has been reported in Soweto, South Africa [8]. Furthermore, whereas high-income countries have seen a decline in the prevalence of high blood pressure, recent prevalence studies conducted across countries in SSA show increasing rates [5, 7, 9, 10]. If the current trends in the prevalence of hypertension remain unchanged, it is projected that by 2025 the number of people with hypertension in the region will increase by 68% (from 74.7 million to 125.7 million) [11].

In Kenya, surveillance data shows that deaths due to NCDs have risen from 35% in 2003 to 45% in 2010 [12] with hypertension being a major contributor to this trend [13]. Studies on hypertension in Kenya have reported prevalence from various settings and populations to range from 18.4 to 32.6% [14–18]. Controlling SBP and DBP is associated with reduction in cardiovascular complications and premature deaths thus impacting the global burden of disease [19, 20]. Increasing awareness, uptake and treatment adherence is an important first step. Between 2003 and 2009, rates of awareness of people with hypertension was 47%, treatment rates were 41% while 32% of individuals on treatment had controlled blood pressure in a multinational population study in 17 countries with urban and rural representation [21]. A recent systematic review involving 33 studies in SSA reported a pooled prevalence of 30% with dismally low rates of awareness (27%), treatment (18%) and control (7%) [7]. Prevalence, awareness and control varies not only between countries but also within countries [8].

In Kenya, while awareness ranged from 6 to 46.5% [15–18], treatment ranged from 9 to 31.3% [14, 16–18] and control levels ranged from 2.3 to 7.4% [14, 16, 18]. Data from the Kenya Demographic and Health Survey (KDHS) 2014 show that only 9.4% of women and 3% of men aged 15–49 years reported that a health care provider had informed them that they have hypertension [22]. Existing prevalence studies in Kenya are based on localized studies that are not generalizable and little is concluded about factors that might influence awareness, treatment or control in these settings. To fill this crucial gap in our understanding, this study examines the prevalence, awareness, treatment, control of hypertension in a nationally representative sample. The results are helpful in providing a baseline for future monitoring and to guiding national policy and programming for better control of hypertension and towards meeting global targets and goals.

## Methods

### Study site and population

Data for this study were obtained from the 2015 Kenya STEPs survey. This was a first of its kind national cross-sectional household survey on NCD risk factors. Data was collected in all 47 counties in Kenya. The aim of the overall study was to provide estimates for indicators on NCD risk factors for persons aged 18–69 years.

### Sample size and sampling

A multistage stratified sampling method was used to allow national estimates by sex (male and female) and residence (urban and rural). The survey used the fifth National Sample Surveys and Evaluation Programme (NASSEP V) master sample frame that was developed by the Kenya National Bureau of Statistics (KNBS). The frame was developed using the Enumeration Areas (EAs) generated from the 2009 Kenya Population and Housing Census to form 5360 clusters split into four equal sub-samples. A total of 6000 households were sampled targeting one individual randomly selected from all eligible household members, of which 4754 gave consent. A total of 4500 eligible individuals were successfully interviewed among the households that gave consent yielding a response rate of 75%. To produce unbiased estimates, sampling weights were calculated as the inverse or reciprocal of all the selection probabilities at all the stages mentioned above. The weights were derived from the processes involved in the creation of sampling frame (NASSEP V) and selection of individuals in the study. Further, the weights were adjusted to cover individual non-responses. Post stratification adjustments were done to align with the population projections according to age-sex categories.

### Data collection

Data were collected by trained personnel using a structured questionnaire adapted from the WHO STEPwise approach to chronic disease risk factor surveillance (STEPS) tool [23] with modifications to suit the Kenyan context. The questionnaire elicited information on demographic characteristics and health behaviors. The field personnel also took blood pressure and heart rate measurements, and anthropometric measurements (height, weight, waist and hip circumference). Data collection occurred between April and June 2015.

Data were recorded on Personal Digital Assistants (PDAs) loaded with eSTEPS software. Further details on the data collection and quality assurance procedures are described elsewhere [24].

### Measurements

Brachial blood pressure and pulse rate were measured using a validated battery powered automated blood pressure machine (OMRON M2 device; Omron Healthcare Co. Kyoto, Japan) with universal cuffs. Blood pressure and pulse rate measurements were taken after a 15 minutes rest while the participant was seated. Three blood pressure and pulse rate measurements were taken 3–5 min apart. The average of the last two blood pressure readings were considered as the final reading for analysis.

Height (in cm) and weight (in Kg) were measured using a scale design with a height gauge (Deluxe GBS-721; Seca Deutschland, Hamburg, Germany).

### Definitions

Hypertension: defined as either having a systolic blood pressure (SBP) equal to or greater than 140 mmHg and/or a diastolic blood pressure (DBP) equal to or greater than 90 mmHg and/or self-report of previous diagnosis of hypertension by a health care provider and/or if currently taking anti-hypertensives in the previous 2 weeks.

Awareness: defined as self-report of prior diagnosis by a health care provider among the participants with hypertension.

Treatment of hypertension: defined as using pharmacologic blood lowering medicines at the time of the interview among those aware of their hypertensive status.

Control of hypertension: defined as SBP below 140 mmHg and DBP below 90 mmHg while on treatment among those on treatment.

Unhealthy intake of fats: defined as self-reported use of saturated fats e.g. lard, margarine, butter and vegetable fat for cooking.

High salt consumption: defined self-report of far too much or too much consumption of actual salt in processed foods, adding salt when cooking and/or to cooked food.

Tobacco use: defined as current use of smoked or smokeless tobacco.

The WHO Global Physical Activity Questionnaire was on used to collect information on physical activity participation [25]. Insufficient physical activity was defined as self-reported less than 150 min of moderate intensive activity or less than 75 min vigorous intensive physical activity per week, including walking and cycling.

Body Mass Index (BMI): computed from the height and weight of the respondent - weight divided by height squared (kg/m^2^). The BMI was further classified into four categories; underweight if BMI was below 18.5 Kg/m^2^, normal if BMI is between 18.50 Kg/m^2^ and 24.99 Kg/m^2^, overweight if BMI is between 25 Kg/m^2^ and 29.99 Kg/m^2^ and obese if BMI is greater than or equal to 30 Kg/m^2^.

Harmful use of alcohol: defined as consumption of more than 1 standard drink (which is the amount of alcohol you find in a small beer, one glass of wine, or one tot of spirits) per day for females and more than 2 standard drinks for males.

### Data analysis

We computed proportions to assess the prevalence, awareness, treatment and control of hypertension. We employed logistic regression models to examine the demographic, behavioral, and body composition factors associated with hypertension and awareness of hypertension. All the known CVD risk factors in the dataset were considered for analysis. First we fitted bivariate logistic regression models to assess the bivariate relationship between the different risk factors and the different outcomes of interest. We then included all the risk factors of interest and those which had a *p*-value of less than 0.25 in the multivariable logistic model. Demographic variables included in the model were age, sex (male or female), education, marital status, place of residence (urban or rural), household socio-economic status, and region. Age was recoded into six categories (18–24, 25–29, 30–34, 35–39, 40–44, 45–49, 50–59, and 60–69). Education was categorized into four categories (no schooling, primary incomplete, primary complete and secondary level and above).

Socio-economic status was measured using a household assets and amenities index that assessed household ownership of various assets (ownership of dwelling and household possessions/goods) and amenities (construction materials of the dwelling, source of water for household consumption, type of sanitation facility and source of cooking oil and lighting fuel) [24]. Standardized weight scores were generated using principal components analysis and ranked to generate wealth quartiles which were recoded into five quintiles with the lowest quintile representing the poorest households to highest quintile representing the wealthiest households [26].

To examine regional differences, we created a categorical variable capturing five geopolitical regions with similar geographical and cultural characteristics; 1) Nairobi and Central (N&C); 2) Coast and North Eastern (C&NE); 3) Eastern (E); 4) Nyanza and Western (N&W), and 5) Rift Valley (RV).

All the analysis were done separately for males and females in addition to an overall model for both. Age-standardized estimates were computed using the 2009 census data to adjust the proportions. Overall *p*-values were used to test the overall effect of a factor. Pairwise *p*-values were used to test the significance of a specific category within a factor with the reference category. *P*-values less than 0.05 were considered to be statistically significant. We used the survey Stata commands (svyset) to account for the study design in order to have nationally representative estimates. All analyses were done using Stata version 15 (Stata Corporation, College Station, TX).

### Ethical approval

The study protocol was reviewed and approved by the Kenya Medical Research Institute’s Ethics Review Committee (SSC No. 2607). All study participants were informed about the study aims including both potential benefits and risks associated with participation. Verbal consent was sought from the household head. All eligible participants gave informed written consent before interview and examination. During the survey, participants who were noted to have abnormalities in their laboratory tests and blood pressure measurements were referred for further care to the nearest health facility or facility of their choice with a referral form. Patient identifiers were delinked from the analytical datasets.

## Results

The analytical dataset used in this study excluded 15 participants with inconsistent age data from the 4500 eligible individuals. An additional 52 participants did not have blood pressure measurements thus a final dataset containing 4433 participants was used for prevalence and multivariable analyses.

The socio-demographic and behavioral characteristics of the study participants are summarized in Tables 1 and 2. Majority (46%) of the participants were young, aged between 18 and 29 years old and 15% were above 50 years of age. Approximately two thirds of the respondents were from the rural areas. More than 60% of the respondents had at least completed primary school education and above. There were significantly more females in rural area as compared to males (*p*-value = 0.013). More females were significantly categorized in the lowest wealth quintile as compared to men (*p*-value = 0.034). Significantly more women had no education as compared to men (*p*-value< 0.001). More men were significantly classified as not in union (*p*-value = 0.029), having normal weight (*p*-value< 0.001), consuming harmful use of alcohol (*p*-value< 0.001), using tobacco (*p*-value< 0.001), and consuming high fat intake (*p*-value< 0.05) as compared to women respectively.

**Table 1:** Participants’ background characteristics by sex

Indicator	Both	Female	Male	*P* value
Age years				
18_24	25.4	25.2	25.5	0.211
25_29	20.6	22.0	19.1	
30_34	12.1	12.7	11.5	
35_39	11.2	11.1	11.3	
40_44	9.4	8.0	10.8	
45_49	6.1	5.4	6.9	
50_59	9.9	10.2	9.5	
60_69	5.3	5.3	5.3	
Residence				
Rural	61.3	64.7	57.8	0.013
Urban	38.7	35.3	42.2	
Wealth status				
Poorest	18.9	21.3	16.4	0.034
Second	20.9	21.8	20.0	
Middle	18.3	18.7	17.8	
Fourth	18.6	16.5	20.7	
Richest	23.4	21.7	25.1	
Education level				
No Schooling	12.6	18.1	6.7	0.000
Primary incomplete	23.3	23.5	23.0	
Primary complete	32.7	34.1	31.3	
Secondary+	31.4	24.3	39.0	
Marital status				
Not in union	34.5	31.9	37.2	0.029
In union	65.5	68.1	62.8	
Province				
Nairobi & Central	28.7	27.7	29.8	0.878
Coast & N Eastern	14.5	15.0	14.0	
Eastern	13.7	14.2	13.1	
Nyanza & Western	22.9	22.6	23.1	
Rift valley	20.2	20.4	20.1	
N	4485	2694	1791

**Table 2:** Participants’ physiological and biological measurements by sex

Indicator	Both	Female	Male	*P* value
Body mass index				
Normal	60.1	52.1	68.1	0.000
Underweight	11.9	9.6	14.3	
Overweight	18.9	24.7	13.2	
Obese	9.0	13.7	4.4	
Harmful use of alcohol	14.4	3.5	25.9	0.000
Insufficient physical activity	7.8	7.5	8.0	0.753
Current tobacco use	13.4	4.1	23.2	0.000
High salt consumption	89.7	89.2	90.2	0.387
High Fat intake	39.7	35.7	43.9	0.002
N	4485	2694	1791	

Out of 4433 participants, 1270 were found to have high blood pressure measurements or on treatment for hypertension. Among the participants with hypertension, 270 were aware of their diagnosis and 136 of those aware of their diagnosis were on treatment while only 50 individuals had achieved blood pressure control among those on treatment. Table 3 summarizes the bivariate age-standardized levels of hypertension, awareness, treatment and control by residence, wealth status, gender, body mass index, behavioral risk factors (alcohol and tobacco use). The overall age-standardized prevalence of hypertension was estimated at 24.5% [95%CI: 22.6–26.6]. Only 15.5% [95%CI: 12.4–18.9] were aware of their condition, 26.9% [95%CI: 17.3–36.4] of the respondents who were aware of their hypertensive status were on treatment for raised blood pressure in the last 2 weeks and 51.7% [95%CI: 33.5–69.9] among those on treatment had their blood pressure controlled.

**Table 3:** Age-standardized hypertension prevalence, awareness, treatment and control by residence, socio economic status and gender

Indicator	Hypertension	Awareness^a^	Treatment^b^	Controlled^c^
	% [95%CI]	% [95%CI]	% [95%CI]	% [95%CI]
National	24.5 [22.6:26.5]	15.5 [12.2:18.7]	26.9 [17.3:36.4]	51.7 [33.5:69.9]
Residence				
Rural	24.7 [22.3:27.2]	12.3 [8.8:15.8]	32.9 [14.2:51.6]	50.2 [29.1:71.3]
Urban	24.9 [21.7:28.1]	20.9 [15.2:26.6]	22.1 [15.8:28.3]	54.6 [37.3:71.9]
Wealth status				
1 - Poorest	19.4 [15.4:23.4]	12.1 [5.6:18.6]	18.4 [4.1:32.8]	81.7 [66.3:97.0]
2	24.3 [20.8:27.9]	15.6 [8.8:22.3]	30.7 [10.7:50.8]	18.4 [12.7:24.1]
3	27.5 [23.1:31.8]	10.8 [6.8:14.8]	17.8 [11.8:23.7]	7.1 [0.5:13.7]
4	24.7 [20.5:28.9]	13.6 [8.8:18.3]	31.5 [15.2:47.9]	61.4 [49.9:73.0]
5 - Richest	29.0 [24.6:33.5]	24.8 [16.0:33.6]	19.9 [13.1:26.7]	40.2 [25.6:54.8]
Sex				
Female	24.2 [22.0:26.4]	22.7 [17.4:28.1]	28.2 [17.2:39.2]	54.2 [33.9:74.4]
Male	25.0 [22.3:27.7]	9.1 [5.4:12.7]	23.6 [8.1:39.2]	62.3 [55.1:69.5]
Body mass index				
Normal	22.0 [20.1:23.9]	10.8 [6.9:14.7]	20.6 [13.9:27.3]	65.1 [57.6:72.7]
Underweight	17.0 [12.3:21.8]	12.7 [4.9:20.4]	66.3 [63.5:69.2]	97.4 [93.4:101.3]
Overweight	30.7 [26.2:35.3]	23.0 [14.1:31.8]	28.6 [12.0:45.1]	37.1 [25.2:48.9]
Obese	42.2 [32.1:52.4]	25.6 [15.2:36.0]	22.0 [11.7:32.4]	18.9 [9.7:28.2]
Harmful use of alcohol				
No	23.6 [21.5:25.8]	15.3 [12.0:18.6]	30.0 [19.0:41.0]	53.6 [35.1:72.1]
Yes	29.2 [23.8:34.5]	15.8 [6.7:24.9]	10.5 [2.5:18.4]	2.5 [−0.8:5.9]
Current tobacco use				
No	24.9 [22.9:26.9]	15.7 [12.4:19.1]	30.9 [18.6:43.2]	50.5 [32.4:68.6]
Yes	24.2 [18.0:30.5]	15.6 [5.2:26.1]	11.9 [5.4:18.4]	16.9 [13.4:20.3]
N	4433	1270	270	136

^a^Among participants with hypertension

^b^Among participants aware of their hypertension diagnosis

^c^Among participants receiving treatment for their hypertension

Hypertension prevalence increased with increasing wealth status; individuals from the richest households had higher hypertension 29.0% [95%CI: 24.6–33.5] compared with those from the poorest households 19.4% [95%CI: 15.4–23.5] and this was statistically significant. Similarly, hypertension prevalence increased with higher BMI; overweight and obese individuals had significantly higher hypertension rates 30.7% [95%CI: 26.2–35.3] and 42.2% [95%CI: 32.1–52.4] respectively compared to underweight individuals 17.0% [95%CI: 12.3–21.8].

Hypertension awareness was significantly higher among individuals in the richest wealth quintile (24.8%) compared to individuals from middle wealth quintile (10.8%), among females (22.7%) compared to males (9.1%) and among the overweight (23.0%) and obese (25.6%) individuals compared to the normal weight individuals (10.8%).

Hypertension treatment was higher among underweight individuals (66.3%) compared to overweight (28.6%) and obese individuals (22.0%), among individuals not consuming harmful amounts of alcohol (30%) compared to those consuming harmful amounts of alcohol (10.5%) and among individuals not currently using tobacco (30.9%) compared to current tobacco users (11.9%).

Blood pressure control was high among the poorest households (81.7%) compared to the richest households (40.2%), among normal weight individuals (65.1%) compared to overweight (37.1%) and obese individuals (18.9%), among those not consuming harmful amounts of alcohol (53.6%) compared to those consuming harmful amounts of alcohol (2.5%) and among non-current tobacco users (50.5%) compared to current tobacco users (16.9%).

The factors associated with hypertension are summarized in Table 4. An increasing positive and significant association of being hypertensive was observed with increasing age and BMI. Respondents older than 50 years old were significantly more than 5 times likely to be hypertensive compared to those aged 18–24 years. BMI was found to be a significant determinant of hypertension; overweight and obese respondents were close to two and three times more likely to be hypertensive as compared to normal weight individuals. Higher BMI was a factor associated with hypertension in both the male and female models. Those consuming harmful amounts of alcohol were 1.54 times more likely to be hypertensive in the overall model however, this effect was mainly from the male model.

**Table 4:** Factors associated with hypertension, overall and by sex

Indicator	Both		Male		Female	
	Adjusted OR	*P*-value*	Adjusted OR	*P*-value*	Adjusted OR	*P*-value*
Age years (Ref: [18–24])	1.00		1.00		1.00	
25–29	1.17 [0.78,1.77]	< 0.001	1.28 [0.73,2.24]	< 0.001	1.02 [0.57,1.83]	< 0.001
30–34	1.24 [0.77,1.99]		1.49 [0.84,2.64]		1.00 [0.51,1.96]	
35–39	*1.99 [1.27,3.11]*		*2.09 [1.11,3.93]*		*1.79 [1.04,3.06]*	
40–44	*2.80 [1.77,4.45]*		*2.59 [1.24,5.41]*		*2.96 [1.57,5.56]*	
45–49	*3.32 [2.16,5.09]*		*2.57 [1.17,5.64]*		*4.16 [2.44,7.09]*	
50–59	*5.41 [3.60,8.12]*		*4.36 [2.33,8.16]*		*6.43 [4.04,10.23]*	
60–69	*8.10 [5.26,12.47]*		*6.18 [3.16,12.08]*		*10.92 [5.91,20.18]*	
Self-reported fat intake (Ref: Good)	1.00		1.00		1.00	
High	1.06 [0.85,1.33]	0.596	1.00 [0.70,1.43]	0.989	1.17 [0.92,1.48]	0.210
High salt consumption (Ref: No)	1.00		1.00		1.00	
Yes	0.86 [0.66,1.12]	0.262	0.85 [0.56,1.30]	0.455	0.89 [0.63,1.25]	0.491
Body mass index (Ref: Normal)	1.00		1.00		1.00	
Underweight	0.71 [0.49,1.03]	< 0.001	0.93 [0.57,1.51]	0.001	*0.49 [0.29,0.83]*	< 0.001
Overweight	*1.73 [1.30,2.30]*		*2.08 [1.31,3.29]*		*1.53 [1.05,2.23]*	
Obese	*2.75 [2.02,3.76]*		*2.27 [1.40,3.69]*		*2.86 [1.81,4.53]*	
Harmful use of alcohol (Ref: No)	1.00		1.00		1.00	
Yes	*1.54 [1.07,2.21]*	< 0.05	*1.65 [1.11,2.47]*	< 0.05	0.93 [0.44,1.97]	0.843
Insufficient physical activity (Ref: No)	1.00		1.00		1.00	
Yes	1.04 [0.73,1.48]	0.834	0.96 [0.55,1.65]	0.873	1.15 [0.77,1.71]	0.503
Current tobacco use (Ref: No)	1.00		1.00		1.00	
Yes	0.91 [0.66,1.26]	0.559	0.85 [0.60,1.20]	0.347	1.46 [0.86,2.50]	0.163
Sex (Ref: Female)	1.00					
Male	1.10 [0.90,1.33]	0.341				
N	4238		1741		2497	

The models adjusted for place of residence, education status, marital status, province and wealth status; *italicized* estimates are significant; *this is the overall *p*-value for the association between the dependent and independent variables.

Table 5 summarizes factors associated with awareness among respondents diagnosed with hypertension in the last 12 months. An increasing positive association of awareness of respondent’s hypertension was observed with increasing age. Individuals aged 60–69 years were significantly close to 4 times likely to be hypertensive compared to those aged 18–24 years. Men had significantly reduced odds of being aware of their hypertensive status [adjusted odds ratio (AOR) = 0.35, (95%CI: 0.22–0.56)] compared to women.

**Table 5:** Results from a multivariable logistic regression on determinants of hypertension awareness among hypertensive respondents, overall and by Sex

Indicator	Both		Male		Female	
	Adjusted OR	*P*-value	Adjusted OR	*P*-value	Adjusted OR	*P*-value
Age years (Ref: [18–24])	1.00		1.00		1.00	
25–29	1.68 [0.55,5.12]	< 0.05	2.61 [0.28,24.21]	< 0.001	1.93 [0.59,6.34]	0.357
30–34	1.21 [0.36,4.10]		*6.73 [1.03,44.16]*		0.66 [0.17,2.57]	
35–39	1.55 [0.52,4.64]		8.07 [0.93,69.73]		0.91 [0.25,3.35]	
40–44	1.25 [0.45,3.52]		0.63 [0.10,3.95]		1.68 [0.52,5.43]	
45–49	2.16 [0.78,5.96]		4.51 [0.35,58.83]		1.92 [0.57,6.52]	
50–59	2.80 [0.94,8.27]		*21.87 [3.89,122.92]*		1.36 [0.40,4.63]	
60–69	*3.75 [1.38,10.23]*		*35.55 [5.97,211.80]*		1.62 [0.53,4.98]	
Self-reported fat intake (Ref: Good)	1.00		1.00		1.00	
High	0.91 [0.59,1.40]	0.666	1.38 [0.57,3.36]	0.475	0.75 [0.45,1.23]	0.251
High salt consumption (Ref: No)	1.00		1.00		1.00	
Yes	0.83 [0.52,1.32]	0.431	1.00 [0.36,2.80]	0.994	0.80 [0.45,1.44]	0.457
Body mass index (Ref: Normal)	1.00		1.00		1.00	
Underweight	1.31 [0.60,2.86]	0.163	1.72 [0.62,4.77]	0.646	0.85 [0.30,2.41]	0.272
Overweight	1.53 [0.87,2.70]		1.10 [0.39,3.09]		1.74 [0.91,3.32]	
Obese	*2.07 [1.08,3.94]*		1.86 [0.28,12.32]		1.96 [0.89,4.29]	
Harmful use of alcohol (Ref: No)	1.00		1.00		1.00	
Yes	1.86 [0.75,4.61]	0.176	2.22 [0.90,5.50]	0.084	0.61 [0.15,2.39]	0.473
Insufficient physical activity (Ref: No)	1.00		1.00		1.00	
Yes	1.72 [0.82,3.60]	0.148	2.38 [0.77,7.38]	0.131	1.50 [0.68,3.31]	0.317
Current tobacco use (Ref: No)	1.00		1.00		1.00	
Yes	0.93 [0.33,2.58]	0.886	0.64 [0.19,2.11]	0.462	1.80 [0.39,8.20]	0.448
Sex (Ref: Female)	1.00					
Male	*0.35 [0.22,0.56]*	< 0.001				
N	1226		484		742	

The models adjusted for place of residence, education status, marital status, province and wealth status; italicized estimates are significant; *this is the overall *p*-value for the association between the dependent and independent variables.

Due to the small number of individuals on treatment (136) and controlled (50), we were unable to run stables regressions for the outcomes of treatment and control.

## Discussion

This study provides important first nationally representative estimates for the burden of hypertension, awareness, treatment and control in Kenya. We further examined factors associated with hypertension and awareness. We found about a quarter of Kenyan adults (18–69 years) were hypertensive. The high prevalence of hypertension is coupled with low hypertension awareness, treatment and control suggesting the need for approaches and interventions that promote prevention, early detection, treatment and control.

### Hypertension prevalence and its determinants

The WHO Africa Region is reported to have the highest prevalence of hypertension (46%) [27]. A previous systematic review and meta-analysis estimated a high prevalence for sub-Saharan Africa (30%) [7]. The current estimate for this study (24.5%) is slightly lower than the prevalence reported for neighboring countries such as Uganda (26.4%) [28] and Tanzania (26%) [29], and much lower than the prevalence reported for Malawi (33%) [30].

Like other studies conducted in similar settings [14, 31–33], we found that the prevalence of hypertension increased with age and was higher among men than women. Similar to other studies [9, 31, 34, 35], the prevalence was higher among urban residents compared to rural. Likely explanations for this include the changes in lifestyle associated with urban living and the presence of widespread risk factors such as harmful use of alcohol, tobacco use, widespread opportunities for unhealthy diets and less opportunities for active living and physical activity. A positive association between hypertension and overweight & obesity was also found in this study. This finding is consistent with findings from other studies [35, 36] and suggests the need to have better targeting of interventions towards patients who are overweight or obese. More attention is required by healthcare workers in the provision of information and health education concerning optimum weight, unhealthy diets, and physical activity. Our study also found an association between alcohol consumption and hypertension as has been found in other studies [14, 37]. Known risk factors for hypertension, such as salt intake, physical activity and tobacco use were found not to be associated with hypertension in this study. A potential explanation for not observing an association is that these measures were self-reported thus it is possible that they may have been reported inaccurately.

### Prevalence and determinants of awareness, treatment and control

Overall, less than a sixth of all individuals with hypertension reported that they had been previously diagnosed with hypertension while most individuals with hypertension in the study were undiagnosed (84.5%), thus a high burden of untreated and uncontrolled hypertension in the Kenyan population. The low levels of awareness are consistent with what has been observed in many SSA countries [7, 14, 28, 38, 39]. We also found higher levels of awareness among older respondents, obese individuals and among female respondents. These findings are consistent with what has been found in several studies [18, 40–42]. Likely explanation for this may be due to higher utilization or more contact with healthcare in these groups. The higher awareness levels in women can be explained by previous studies that have shown women reporting greater use of health services than men [43–45]. This result has implications on hypertension care programming in Kenya; highlighting the need to have more target specific campaigns and opportunities for screening and treatment for men.

Treatment levels were very low in Kenya and reflect the low awareness levels found in this study. Costs associated with treatment continue to pose a challenge to hypertension treatment especially among individuals aware of their hypertensive status. The 2003 Kenya Household Health Expenditure and Utilization Survey shows that 21.4% of the respondents did not seek treatment to illness due to high cost [45]. Universal healthcare coverage or having health insurance might be important in improving access to care. A study conducted in Namibia showed that better blood pressure control was achieved by those who were insured [16]. Unlike most studies [14, 38, 40, 42], we didn’t find a statistical difference in having more women on treatment compared to men.

While it is important to increase treatment levels, even more important is to examine whether the treatment being received is effective. High blood pressure is an established leading contributor to the development of cardiovascular disease (CVD) [46]. Treating high blood pressure is associated with a reduction in cardiovascular complications and premature deaths [47, 48]. Conversely, uncontrolled hypertension can lead to adverse cardiovascular outcomes [48]. Among all those on treatment in our study, a little over 50% of the study participants on treatment had achieved the target blood pressure. This rate ideally should be close to 100% but the inability to achieve it could stem from many factors including widespread use of counterfeit drugs. Although it is difficult to estimate the levels of counterfeit drugs, previous studies have reported high levels of counterfeit drugs in SSA. Published estimates of counterfeits vary from 1 to 50% of all pharmaceuticals [49]. WHO has estimated that 50% of essential drugs in Africa are counterfeit [50]. Other likely reasons could include poor adherence to medication due to side effects, and therapy with only 1 BP pill when therapy with more than 1 BP pill is necessary. Findings from a large multinational study revealed the use of more than one medicine to treat hypertension was significantly lower in low-income countries compared the higher-, upper middle- or the lower middle-income countries [21].

### Strengths and limitations

This study has both strengths and limitations. A key strength of the current study is the large sample size with national geographic coverage to provide estimates that can be extrapolated to the general population. Blood pressure measurements were taken in a single visit hence the burden of high blood pressure could have been overestimated. Sensitivity analysis conducted in a previous study in sub-Saharan Africa suggests an overestimation of 5.4% among those unaware of their hypertension status previously [32] while a more recent study reported an overestimation of 23.4% [51]. Another important limitation is the cross-sectional nature of the study ruling out any causal association. Dietary salt intake, fat intake and physical activity measures were based on self-reported information, which is subject to bias and can lead to incorrect estimates. A key limitation is the missed opportunity of the current study to collect medication use data that could explain the treatment levels.

## Conclusions

This is the first national survey on hypertension to be conducted highlighting hypertension as a major health problem in Kenya. This survey is important and gives a national picture of the hypertension situation which has been lacking to guide policies and interventions. The study results show a high hypertension prevalence coupled with low awareness and treatment. The high level of undiagnosed hypertension coupled with low levels of treatment can result in adverse cardiovascular outcomes that are costly to treat and this can potentially overburden the already over-stretched health care system. These results support an urgent need to develop new strategies, policies and programs that will promote prevention, increase screening, as well as expand access and adherence to effective treatment. Results from this national study are important as they highlight lower awareness of hypertension among males. This sex differences underscore the need for integrated programs that address men’s needs for information and services relating to hypertension prevention and treatment.

Also, given that this study is embedded in the first national NCD risk factor survey in Kenya, there should be concerted efforts to have regular population data surveys for NCD risk factors so that trend data can better inform and update the policies, programs and interventions.

## Abbreviations

CVD: Cardiovascular disease
DBP: Diastolic blood pressure
KEMRI: Kenya Medical Research Institute
NCD: Non-communicable diseases
SBP: Systolic blood pressure
SSA: Sub-Saharan Africa
